# A quantitative study of the effect of ICL orientation selection on post-operative vault and model-assisted vault prediction

**DOI:** 10.3389/fneur.2023.1136579

**Published:** 2023-03-03

**Authors:** Weijie Zhang, Fang Li, Lin Li, Jing Zhang

**Affiliations:** ^1^Department of Ophthalmology, Ninth People's Hospital, Shanghai Jiao Tong University, School of Medicine, Shanghai, China; ^2^Shanghai Key Laboratory of Orbital Diseases and Ocular Oncology, Shanghai, China

**Keywords:** ICL implantation orientation, vault height, machine-learning, optic nerve, myopia

## Abstract

**Background:**

Appropriate vault height of implantable collamer lens (ICL) implantation matters for it has risks of corneal endothelial cell loss, cataract formation and intraocular pressure elevation, which could lead to irreversible damage to optic nerve. Therefore, pre-operative prediction for an ideal vault height is a hotspot. However, few data exist regarding quantitative effect of ICL orientation on vault height. This study is aimed to quantitatively investigate the effect of ICL implantation orientation on vault height, and built a machine-learning (ML)-based vault prediction model taking implantation orientation into account.

**Methods:**

473 consecutive case series treated with ICL implantation were retrospectively analyzed (408 were horizontally implanted, and 65 were vertically implanted). Multivariable logistic regression analysis was performed to determine the association between ICL orientation and achieved vault. ML was performed to develop a new vault height prediction model taking ICL orientation into account. Receiver operating characteristic curve (ROC) and net reclassification index (NRI) were obtained to assess the prediction ability.

**Results:**

95% of all the patients achieved 20/20 uncorrected distance visual acuity (UDVA) or better. No complications including cataract formation, dispersion or optic nerve injury were observed in any cases. Sex, sphere power, cylinder power, axis, ICL size and ICL orientation were all significant risk factors associated to vault height, and age was positively co-related. Of note, ICL size and ICL orientation were the top-ranking risk factors. Comparing to conventional horizontal implantation, vertical implantation could reduce the achieved vault by 81.187 μm (*p* < 0.001). In regarding to different ICL sizes, vertical implantation had no good to vault reduction when using ICL of 12.1 mm. However, it could reduce the vault by 59.351 μm and 160.992 μm respectively when ICL of 12.6mm and 13.2 mm were implanted (*p* = 0.0097 and *p* = 0.0124). For prediction of vault height, ML based model significantly outperformed traditional multivariable regression model.

**Conclusion:**

We provide quantitative evidence that vertical implantation of ICL could effectively reduce the achieved vault height, especially when large size ICL was implanted, comparing to traditional horizontal implantation. ML is extremely applicable in development of vault prediction model.

## Introduction

The implantable collamer lens (ICL) has been widely recognized as a safe, predictable, and efficient strategy for refractive correction, especially with moderate-to-high refractive errors or corneal contraindications (1–4). Nevertheless, the main concerns focusing on the potential risk of cataract formation (2, 4–7), corneal endothelial cell loss (4, 6), pigment dispersion and related glaucoma (8), pupillary block glaucoma (9, 10), are all highly related to the ICL vault height (11–13). Therefore, an ideal post-operative vault height is critical for the safety of ICL implantation and an important assessment parameter during the follow-ups.

Previous studies have indicated that ICL size was the one of the most important parameters affecting post-operative vault height (12). The ICL is designed to be fixed in the ciliary sulcus, which could provide stability and appropriate compression force to achieve proper vault height. When the difference between the ciliary sulcus size and ICL size was too big, the relatively oversized ICL led to excessive vault. On the contrary, an undersized ICL could lead to insufficient vault. However, there are only four sizes of ICL for selection: 12.1, 12.6, 13.2 and 13.7 mm. Obviously, the discontinuity of ICL sizes for selection means that complete customization cannot be achieved clinically, because the anterior segment parameters of patients continuously distributed, which is consistent with the clinical observation, that unacceptable post-operative vault was obtained in some cases. To date, ICL exchange has been a routine method to address oversized ICL or related high vaults. In 2018, a case report first claimed that rotating the implanted ICL from horizontal to vertical orientation effectively declined the achieved vault, providing a new, much less invasive and more economical choice (14). A recent retrospective case series also proved that apart from ICL size, the implantation orientation also resulted in different vault outcomes (15). These studies inspired us to explore further detailed questions: How much could this simple change on implantation angle affect the achieved vault height? Does it contribute to a more satisfactory vault height (250~750 μm) (16, 17)?

Therefore, the aim of this study is to quantify the vault height difference between the two strategies for ICL implanting angle. Moreover, a new Machine-learning (ML) -based prediction model for vault height is developed referring to the implantation orientation.

## Materials and methods

Patients who underwent ICL-V4C implantation at Shanghai Ninth People's Hospital in Shanghai, China between January 2019 and August 2020 were enrolled in this continuous retrospective series, and the follow-up was 12 months. All surgeries were conducted by one surgeon.

This study adhered to the tenets of the Declaration of Helsinki, and ethical approval was obtained from/ and is approved by the Ethics Committee of Shanghai Ninth People's Hospital (SH9H-2021-T278-1).

Inclusion criteria are as follows: age older than 18 years; myopia ≤ −18.0D, astigmatism ≤ 6.0D, stable for at least 1year (defined as progression in refraction of no more than −0.5D per year); corrected distance visual acuity (CDVA) ≥ 20/40; anterior chamber depth (ACD) > 2.8 mm; and central corneal endothelial cell count ≥ 2000 cells/mm^2^.

Exclusion criteria are: history of eye surgeries; trauma; other ophthalmic diseases including cataract, glaucoma, uveitis, retinal detachment, retinal pigment degeneration, corneal dystrophy and corneal endothelial dysfunction etc.; uncontrolled systemic diseases like systemic lupus erythematosus, multiple sclerosis, rheumatoid arthritis, and severe diabetes mellitus; or other reasons that could affect following measurement such as abnormal mental behavior and so on.

### Preoperative evaluation

A comprehensive ophthalmic examination was conducted before ICL implantation, including uncorrected distance visual acuity (UDVA), auto refraction, manifest refraction, anterior segmental examination and fundus examination using slit lamp microscope, and intraocular pressure (IOP) measured by noncontact tonometer (CT-80; Topcon, Tokyo, Japan). The ACD was measured using IOL Master 500^®^ (Carl Zeiss, JeTna, Germany). The endothelial cell density (ECD) was measured by non-contact specular microscope (SP-2000P; Topcon, Tokyo, Japan). Horizontal white-to-white distance (WTW) and central corneal thickness (CCT) were measured with Pentacam HR (OCULUS, Wetzlar, Germany).

The ICL power calculation was performed according to the manufacturer's instructions using all related parameters, and the surgical goal in all eyes is to achieve emmetropia. For ICL sizing, the recommended STAAR nomogram was used based on WTW and ACD parameters. The lens model used in this study was non-toric ICL V4c. Conventional horizontal angle was adopted for the first eye. For the second eye, the ICL implantation angle was decided according to the achieved vault of the first operated eye. To be specific, when the achieved vault height of the first eye was relatively excessive but still within the satisfactory vault height range (250 to 750 μm, we chose vertical implantation for the second eye of the same patient.

### Surgical procedure

Compound topicamide eye drops were applied every 10 min to fully dilate the pupils before surgery. After topical anesthesia with Oxybuprocaine Hydrochloride Eye Drops, a clear 3.2 mm corneal incision was made, and sodium hyaluronate was injected into the anterior chamber. The ICL lens was implanted in the posterior chamber and aligned horizontally or vertically, with a special injection device provided by the manufacturer. The sodium hyaluronate was removed completely by irrigation/aspiration process. Pupils were constricted immediately by administration of pilocarpine eye drops after surgery. To bradex eye drops containing 0.3% wt/vol tobramycin and 0.1% wt/vol dexamethasone were used 3 times daily for 1 week, and then tapered off over the next 2 weeks. Additional single glaucoma medication was topically applied according to the judgement of clinicians.

### Follow-ups

Follow-up assessments were performed 1 day, 1 week, 1, 3, 6 and 12 months after the surgery. CDVA, UDVA, and IOP were recorded with the aforementioned methods. The vault height was measured using Pentacam HR.

### Prediction model of the vault height

The achieved vault heights were artificially divided into two groups: optimal vault heights (250 μm~750 μm) and sub-optimal vault heights (<250 μm or more than 750 μm). There are two cohorts in our study: (1) training cohort including patients who underwent surgery from January 2019 to December 2019, and (2) validation cohort including patients who underwent surgery from January 2020 to August 2020. The quantity ratio between the training set and the validation cohort was 85:15. In the training cohort, we firstly constructed the multivariable logistic regression model with 10-fold cross-validation. Meanwhile, the random forest (RF) algorithm, which is an important ML algorithm that adopts a bootstrapping resampling technique and selects feature sets *via* random sampling and random selection, was also employed in this study.

The classification performance of the above models was evaluated in the validation cohort. Firstly, the confusion matrix analysis of the two prediction models were conducted. To further assess the performance of these two models, the receiver operating characteristic curve (ROC) analysis was conducted to calculate the area under the ROC curve (AUC). Moreover, NRI was also used to evaluate the classification efficacy of the two prediction models.

### Statistical analysis

All results were expressed as mean ± standard deviation for continuous parametric data, and percentages for categorical data. A stepwise multivariable logistic regression analysis was used to investigate the meaningful variants relevant to the vault height, and the difference of the vault height between two implantation orientations were calculated according to the coefficients. Statistical analysis in this study were performed in R (version 4.1.1; R Foundation for Statistical Computing, Vienna, Austria; http://www.r-project.org). A value of *P* < 0.05 was considered significant.

## Results

### Baseline characteristics

A total of 473 consecutive eyes were retrospectively enrolled in this study; the baseline characteristics are provided in Table 1. Of them, 408 eyes (86.26%) underwent horizontal ICL implantation, and 65 eyes (13.74%) underwent vertical ICL implantation. The mean age of the patients was 28.41 ± 6.22 years, and 72.73% (344/473) of the patients were male. The mean preoperative sphere and cylinder power were −8.64 ± 2.86 diopters and −1.27 ± 1.14 diopters, respectively. The number of eyes implanted with different ICL sizes was 78 for 12.1 mm, 302 for 12.6 mm, 85 for 13.2 mm and 3 for 13.7 mm, as shown in Table 1. We also listed the clinical characteristics grouped according to different sizes of the implanted ICL. Other biometrics including WTW, CCT and ECD were also shown in Table 1.

**Table 1:** Preoperative clinical characteristics, ICL orientation and vault height of patients undergoing ICL implantation.

	**ICL size = 12.1 mm (*N =* 78)**	**ICL size = 12.6 mm (*N =* 302)**	**ICL size = 13.2 mm (*N =* 85)**	**ICL size = 13.7 mm (*N =* 8)**	**Overall (*N =* 473)**
Age (years)	29.46 ± 6.14	28.58 ± 6.39	26.96 ± 5.47	28.00 ± 6.92	28.41 ± 6.22
Sex (male, %)	58/78 (74.36%)	225/302 (74.50%)	61/85 (71.76%)	0/8 (00.00%)	344/473 (72.73%)
Sphere (diopters)	−9.49 ± 2.96	−8.46 ± 2.85	−8.50 ± 2.73	−9.92 ± 1.13	−8.64 ± 2.86
Cylinder (diopters)	−1.08 ± 0.94	−1.28 ± 1.14	−1.35 ± 1.11	−0.41 ± 0.38	−1.27 ± 1.14
Axis (degree)	99.64 ± 76.96	91.77 ± 77.42	103.35 ± 78.14	78.33 ± 70.05	94.7 ± 77.32
WTW (mm)	11.12 ± 0.17	11.62 ± 0.21	12.09 ± 0.22	12.63 ± 0.05	11.64 ± 0.36
ACD (μm)	3.09 ± 0.21	3.19 ± 0.22	3.37 ± 0.18	3.44 ± 0.05	3.22 ± 0.23
CCT (μm)	526.96 ± 37.5	525.47 ± 32.11	530.68 ± 37.32	517.66 ± 22.94	526.32 ± 34.09
ECD (cell/mm^2^)	2705.94 ± 413.93	2819.40 ± 370.77	2844.68 ± 412.44	2701.66 ± 255.97	2804.87 ± 386.07
lCL orientation (vertical, %)	4/78 (5.13%)	43/302 (14.24%)	13/85 (15.29%)	5/8 (62.50%)	65/473 (13.74%)
Vault height (μm)	309.15 ± 74.48	350.95 ± 121.93	455.32 ± 177.63	345.00 ± 31.22	362.52 ± 135.04

WTW, white-to-white distance; ACD, anterior chamber depth; CCT, central corneal thickness; ECD, endothelial corneal density.

### Outcome and complications

ICL lenses were implanted at one time, and no lens replacement or second operation occurred in any of the eyes. Transient IOP elevation was observed in four patients, which returned to normal at the next follow-up visit after topical application of single glaucoma medication. There was no sign of cataract formation, pigment dispersion, or other ICL-related complications especially optic nerve injury in any of the eyes during the follow-ups.

At the end of the follow-up, 95% of all the patients achieved 20/20 UDVA or better, which is significantly improved comparing to the preoperative UDVA. 85% of all the patients had a post-operative manifest refraction of no more than ± 0.5 diopters.

The mean post-operative vault height at the last follow-up was 362.52 ± 135.04 μm shown in Table 1. For different ICL sizes, the mean achieved vault height was also listed.

### Multivariable logistic regression analysis of parameters relating to vault height

With the use of pre-operative parameters as independent variables, a stepwise multivariable logistic regression analysis was performed to determine meaningful factors affecting the achieved vault height.

As shown in Table 2, sex, sphere power, cylinder power, axis, ICL size and ICL implantation orientation were all independent risk factors for vault height (*p* < 0.01). Age acted as a protective factor against excessive vault height (*p* = 0.01727).

**Table 2:** Coefficient measures of each of the parameters and the achieved vault height.

	**Sex**	**Age (Years)**	**Sphere 2 (diopters)**	**Sphere 3 (diopters)**	**Sphere 4 (diopters)**	**Cylinder2 (diopters)**	**Cylinder3 (diopters)**	**Axis (degree)**	**WTW (mm)**	**ACD (μm)**	**CCT (μm)**	**ECD (cell/mm^2^)**	**ICL type**	**ICL size = 12.6mm**	**ICL size = 13.2mm**	**ICL size = 13.7mm**
**Total**																
Estimate Std.	45.512	−2.564	218.394	189.069	200.333	−12.586	54.831	0.01	−71.461	36.404	0.053	−0.023	−5.099	54.053	198.715	189.739
Error	14.882	1.072	67.968	65.511	65.718	19.528	31.217	0.086	46.449	29.716	0.193	0.018	21.645	24.011	37.608	89.188
t value	3.058	−2.391	3.213	2.886	3.048	−0.645	1.756	0.122	−1.539	1.225	0.274	−1.269	−0.236	2.251	5.284	2.127
Pr(>|t|)	0.002^**^	0.002^*^	0.001^**^	0.001^**^	0.002^**^	0.52	0.08	0.903	0.125	0.221	0.784	0.205	0.814	0.020^**^	0.000^***^	0.034^*^
**ICL size** = **12.1 mm**																
Estimate Std.	43.762	-1.200		57.626	55.505	−31.858	−9.89	0.299	11.77	−36.081	0.241	−0.062	15.54			
Error	23.121	1.568		73.407	76.112	30.157	51.763	0.127	82.192	54.571	0.282	0.027	31.204			
t value	1.893	−0.766		0.785	0.729	−1.056	−0.191	2.367	0.143	−0.661	0.855	−2.281	0.498			
Pr(>|t|)	0.065	0.448		0.436	0.469	0.296	0.849	0.022*	0.887	0.512	0.397	0.027^*^	0.621			
**ICL size** = **12.6 mm**																
Estimate Std.	35.063	−2.776	162.627	130.798	155.061	−7.47	44.562	0.032	−93.185	38.246	−0.092	−0.053	−5.522			
Error	17.318	1.319	86.796	84.609	84.224	21.935	35.001	0.099	50.51	34.509	0.237	0.022	23.795			
t value	2.025	−2.104	1.874	1.546	1.841	−0.341	1.273	0.326	−1.845	1.108	−0.387	−2.358	−0.232			
Pr(>|t|)	0.044^*^	0.044^*^	0.062	0.123	0.067	0.734	0.204	0.745	0.066	0.269	0.699	0.019	0.817			
**ICL size** = **13.2**																
Estimate Std.	62.091	−9.668	24.237	99.766	125.102	27.504	101.062	−0.257	−126.285	156.766	0.86	0.066	18.276			
Error	57.664	4.577	175.492	156.655	157.854	88.193	119.023	0.293	164.972	125.715	0.642	0.057	111.421			
t value	1.077	−2.112	0.138	0.637	0.793	0.312	0.849	−0.877	−0.765	1.247	1.34	1.156	0.164			
Pr(>|t|)	0.286	0.039*	0.891	0.527	0.431	0.756	0.399	0.384	0.447	0.217	0.186	0.252	0.87			

Myopia was graded as four levels according to sphere power: level 1 (>-300 diopters), level 2 (−300 to −600 diopters), level 3 (−600 to −1200 diopters) and level 4 (more than −1200 diopters). Astigmatism was graded as three levels according to cylinder power: level 1 (less than 100 diopters), level 2 (100 to 200 diopters), level 3 (more than 200 diopters). Sphere 2 = sphere diopters of level 2– sphere diopters of level 1; Sphere 3 = sphere diopters of level 3– sphere diopters of level 2; Sphere 4 = sphere diopters of level 4– sphere diopters of level 3. Cylinder 2 = cylinder diopters of level 2-cylinder diopters of level 1; Cylinder 3 = cylinder diopters of level 3-cylinder diopters of level 2.

* P < 0.05,

** P < 0.01,

*** P < 0.001.

**Figure 1:**
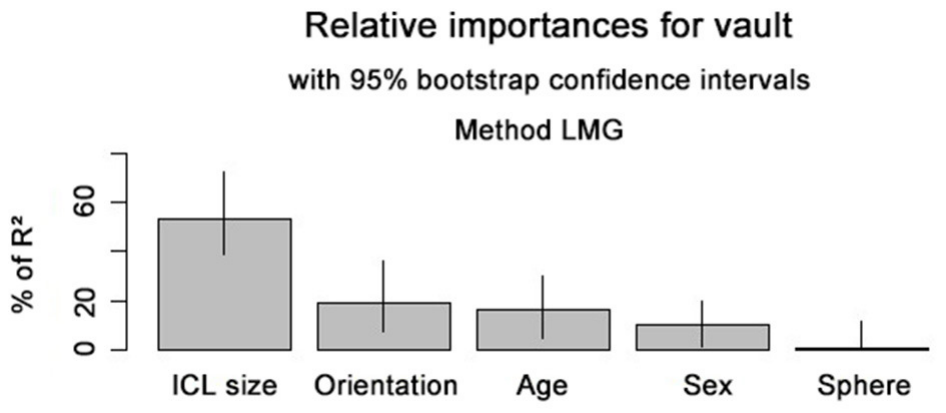
Rank of risk factors relating to vault heigh.

We also ranked the importance of these risk factors according to the coefficient of determinations for the achieved vault height (shown in Figure 1). Results showed that ICL size and orientation of ICL implantation were two most important risk factors associated with vault height, and age, sex, and sphere power were also closely related to it.

For ICL sizes of 12.1 mm, implantation orientation ranked only second to the ICL size. Notably, for ICL size of 12.6 and 13.2 mm, implantation orientation mattered the most (shown in Supplementary Figure 1).

### Comparison of general clinical characteristics between the vertical and horizontal implantation groups

We compared the clinical characteristics between the two different implantation orientations (horizontal or vertical). No statistical difference was found in age, sex, WTW, ACD, ECD, CDVA, and IOP between the vertical-implantation group and horizontal-implantation group. Specifically, for patients implanted with ICL size of 12.1 mm, 12.6 mm and 13.2 mm, the above-mentioned parameters also showed no significant difference (*p* > 0.05).

### Contribution of ICL orientation to the achieved vault height

Previous studies proved that when larger-sized ICL was used, vertical implantation could efficiently reduce the vault height compared to horizontal implantation (15). In this study, we further quantitatively explored to what extent the ICL implantation orientation could affect the vault height. As shown in Table 2, a mean decrease of 81.18744 μm is achieved by implantation with a vertical orientation compared to horizontal orientation when other parameters were adjusted (*p* < 0.001). We also conducted subgroup regression analysis for different ICL sizes, and obtained the following results: (1) for ICL size of 12.1 mm, the implantation orientation is not an independent risk factor for increase of vault height (*N* = 78, *p* = 0.5665); (2) for ICL size of 12.6 mm, the implantation orientation is an independent risk factor for excessive vault height (*N* = 302, *p* = 0.0097); and after adjusting for other risk factors, vertical placement achieved a mean vault decrease of 59.351 μm comparing to horizontal placement; (3) for ICL size of 13.2 mm, the implantation orientation also acts as an independent risk factor for excessive vault height (*N* = 85, *p* = 0.00124 and a mean vault decrease of 160.99214 μm of vault height is obtained when ICL is implanted vertically; (4) for ICL sizing of 13.7 mm, the subgroup analysis was not completed due to the small sample size (*N* = 8).

### Prediction model for vault height

We next established prediction models for vault height with multivariable regression analysis and machine-learning respectively, based on the meaningful biometric data in the training-set. Then the predictability of the two models were examined based on the data in the validation cohort. In the validation cohort with a sample size of 60, 57 is defined as positive, which means the post-operative vault height lies in the range of ideal vault (250 to 750 μm); and 3 is defined as negative, with a low or high vault height. No information rate (NIR) is 0.95 for this data set. The performance of the two models for post-operative vault height prediction was assessed statistically (Table 3).

**Table 3:** Basic comparison between two prediction models.

	**Logistic regression**	**Random forest**
Accuracy	0.967	1.000
95% CI	(0.885, 0.996)	(0.940, 1.000)
Sensitivity	0.333	1.000
Specificity	1.000	1.000
PPV	1.000	1.000
NPV	0.966	1.000

PPV, positive prediction value; NPV, negative prediction value.

**Figure 2:**
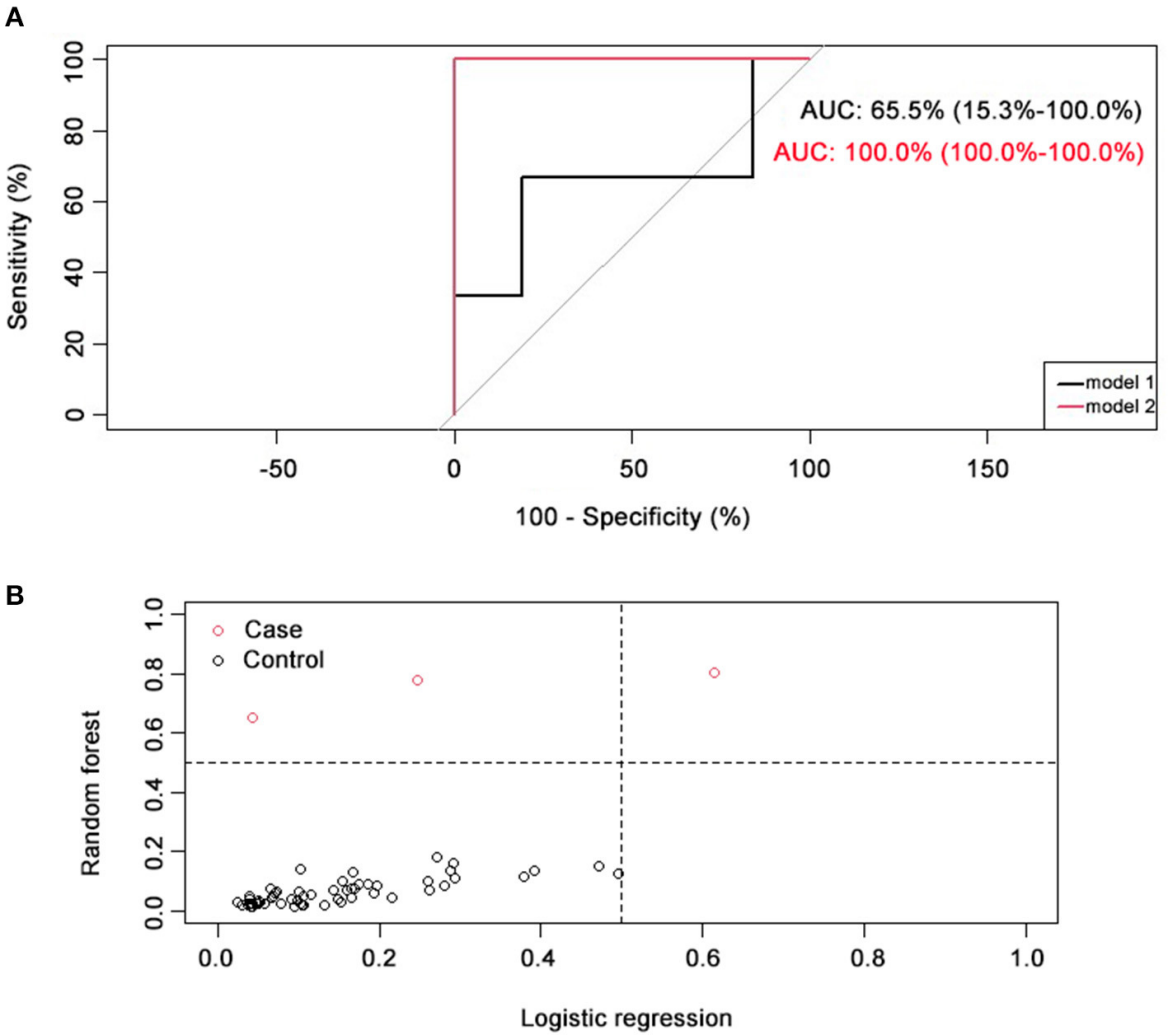
Comparison of two prediction models. **(A)** ROC curve comparison of two prediction models. AUC for logistic regression model (model 1, black) was 65.5% (15.3~100.0%), and AUC for random forest model (model 2, red) was 100.0% (100.0~100.0%). **(B)** Classification capacity of two prediction models. NRI = 0.67 (0.1). Positive cases (with an optimal vault height) were represented with black circles, and negative cases (with a sub-optimal vault height) were represented with red circles.

ROC curve regression analysis was performed to directly compare the discrimination ability of the two models (shown in Figure 2A). Results showed that the model based on machine-learning had a greater AUC (AUC = 100%) than the regression model (AUC = 65.5%).

To further compare the classification efficacy of these two models, NRI was calculated, and results showed that NRI = 0.67 (0.1), indicating the priority of the machine-learning-based model comparing to traditional regression model (shown in Figure 2B).

## Discussion

This study conducted a quantitative analysis for the first time on the influence of ICL implantation orientation on the post-operative vault height. Moreover, a ML-based vault height prediction model has also been developed, taking the implantation orientation into account, and exhibited a satisfactory performance.

Clinically, the online formula used for ICL sizing could not always achieve a satisfactory vault height, and many newly developed prediction models are not applicable in most clinics. Previous studies have proved that vertical ICL implantation in the ciliary sulcus could provide more space for the implanted lens comparing to horizontal implantation, thus resulting in lower vault height (14, 15). On this basis, our study further identified that the difference of vault height between the two implantation angles was 81.187 μm. We also conducted subgroup regression analysis for different ICL sizes, and results showed that vertical implantation could reduce the vault height by 59.351 μm and 160.992 μm respectively when ICL of 12.6 mm and 13.2 mm were implanted. It is meaningful for guiding clinical practice, especially in second surgery for patients with inappropriate vault height, and surgeons could roughly estimate the achieved vault height obtained by fine-tuning the implantation orientation. Moreover, a ML-based vault height prediction model has also been developed, taking the implantation orientation into account, and exhibited a satisfactory performance.

With the development of surgical procedures and accumulation of clinical experience, ICL implantation has been widely recognized as a safe and efficient strategy for refractive correction (1). Therefore, number of studies on visual quality gradually decreased, while prevention of complications has drawn increasing attention. The major post-operative complications include cataract formation, angle-closure related glaucoma, pigment diffusion of iris, and corneal endothelial dysfunction, which are all associated with inappropriate vault height (11). Therefore, one hotspot for discussion is about the acceptable or ideal vault height, which has been studied from various perspectives.

Early researchers roughly measured the vault height semi-quantitatively by comparing with CCT through slit lamp examination, and an initial consensus is that a vault value close to the CCT is acceptable, which is also the targeting vault of the STAAR nomogram when recommending ICL sizes (18). Later studies further suggested an ideal vault range of 30% to 100% of the CCT (12). This measurement approach, i.e., comparison with CCT, is based on the fact that the average CCT is generally 500 μm. However, CCTs in general population are Gaussian distributed, and previous standards are obviously not applicable for those with too thin or too thick corneas. With development of vault measurement methods such as ultrasound biomicroscopy (UBM) and optical coherence tomography for anterior segment (AS-OCT) (16, 19–22), researchers began to evaluate the vault with objective absolute value, and usually an ideal vault was determined as 250–750 μm (16, 17). Previous study (23) also indicated that there was strong congruence for vault value among different evaluation approaches. In this study, we assessed the absolute value post-operative vault height with Pentacam HR (Table 1). Our result suggested that CCT was not a risk factor in the prediction of vault height (Table 2).

Previous study proved that the post-operative vault value gradually decreased with the prolongation of follow-up time (24). Generally, the vault value gradually decreased in the first 3 to 6 months after surgery, and stabilized at the 6 to 12 months, which is the reason why many studies chose 6 or 12 months as the follow-up time. In our study, the cases were followed for 12 months.

Nowadays, different ML algorithms and the introduction of various parameters have been widely applied in the refined diagnosis of ophthalmic diseases (25–31) and prediction of efficacy of therapeutic regimens (32, 33).

In relation to ICL sizing calculation or vault height prediction, clinicians usually adopt the STAAR nomogram based on the WTW formula measured by Pentacam HR, which is recommended by the manufactures. Recently, new formulas were developed by different research teams, such as the KS (19) and NK models (16, 34). New parameters besides WTW and ACD were also introduced including sulcus-to-sulcus diameter (STS), crystalline lens rise (CLR), angle-to-angle (ATA), distance between STS plane and anterior crystalline lens surface (STSL) etc. (22, 35–39). Theoretically, the introduction of more parameters contributes to the delicate description of the anterior segment. However, large-scale clinical trials are needed to verify the efficacy and priority of these formulas before application in usual clinical practice. Basically, there are two limitations in the development of these clinical studies: the accessibility of examination instruments and the improvement space for clinicians.

Traditional parameters like ACD and WTW, can be measured by IOL Master and Pentacam HR, which are allocated in many ophthalmic clinics. However, new parameters like STS, CLR, ATA, STSL can only be obtained with high-resolution UBM or AS-OCT, which are expensive and are not accessible in most centers. What's more, a comparative study showed that data collected by Pentacam HR, AS-OCT and UBM could not replace each other due to different measuring principles (21), especially when applied in vault prediction (40). In addition, there is also controversy regarding the superiority of STS over WTW in ICL sizing and vault prediction (41). Moreover, the application of UBM is also restricted as a contact examination, especially during current period with high incidence of infectious disease. These findings signified the application value of our study.

In this study, the pre-operative parameters are covariates and cannot be changed. For the operators, this means that the potential manipulation space for further post-operative improvement is very limited. Our study provides an additional choice to improve the postoperative results, and helps clinicians to quantitatively estimate the change of the vault height obtained by changing the implantation orientation of ICL. Results demonstrated that a significant vault height decrease of 59.351 μm and 160.992 μm were achieved respectively, when implanted with ICL sizes of 12.6 mm and 13.2 mm vertically. However, no significant difference in the vault height was observed when implanted with ICL of 12.1 mm between the two implantation angles. Probable explanation is that if the ICL size is small, the horizontal sulcus imposes a relatively small compression force on the ICL lens; as a result, larger distance achieved by rotation has little influence on the ICL morphology and dynamics, as well as the achieved vault height.

Two approaches for vault height prediction were applied in this study: multivariable logistic regression analysis and ML, and the prediction ability of the two models were assessed by comparing ROC and NRI. Results showed that in most cases, ICL implantation achieved an ideal vault height. Moreover, the ML-based prediction model showed significant priority in minimizing the incidence of sub-optimal vaults.

There are some limitations for our study. Firstly, the 473 cases derived from 473 eyes of 238 patients after eliminating the missing data. We believe further studies should be conducted on single eye of each patient to minimize bias. Another limitation is that our study is a retrospective study, and the result can only suggest that ICL implantation angle correlates with the post-operative vault height. Further evidence of causal relationship should be provided by interfering the ICL placement in large-scale RCT researches.

## Data availability statement

The original contributions presented in the study are included in the article/Supplementary material, further inquiries can be directed to the corresponding author/s.

## Ethics statement

The studies involving human participants were reviewed and approved by Ethics Committee of Shanghai Ninth People's Hospital (SH9H-2021-T278-1). The patients/participants provided their written informed consent to participate in this study.

## Author contributions

WZ: writing – original draft, methodology, and project administration. FL: software, data analysis, and project administration. LL: writing – review and editing, data analysis, and supervision. JZ: conceptualization, resources, writing – review and editing, and supervision. All authors contributed to the article and approved the submitted version.
