# Supplementary figures and images for: A quantitative study of the effect of ICL orientation selection on post-operative vault and model-assisted vault prediction
Source: Front Neurol. 2023 Mar 3;14:1136579. doi: 10.3389/fneur.2023.1136579 (PMC10020497; doi:10.3389/fneur.2023.1136579)

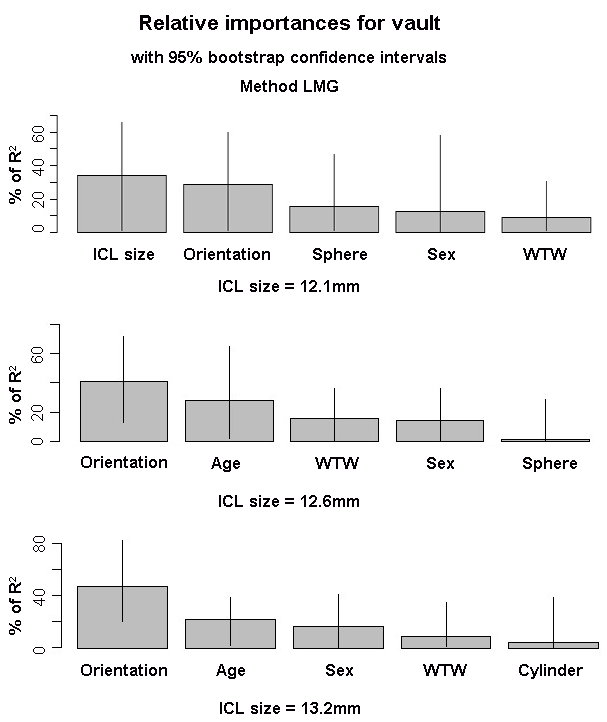

Supplement: Supplementary file 1 [file Image_1.TIF]
